# Disrupted Ankle Control and Spasticity in Persons With Spinal Cord Injury: The Association Between Neurophysiologic Measures and Function. A Scoping Review

**DOI:** 10.3389/fneur.2020.00166

**Published:** 2020-03-11

**Authors:** Jasmine M. Hope, Ryan Z. Koter, Stephen P. Estes, Edelle C. Field-Fote

**Affiliations:** ^1^Shepherd Center, Crawford Research Institute, Atlanta, GA, United States; ^2^Graduate Division of Biological and Biomedical Sciences, Laney Graduate School, Emory University, Atlanta, GA, United States; ^3^Division of Physical Therapy, School of Medicine, Emory University, Atlanta, GA, United States; ^4^Georgia Institute of Technology, School of Biological Sciences, Program in Applied Physiology, Atlanta, GA, United States

**Keywords:** corticospinal tract, spinal reflex circuit, Hoffman-reflex, transcranial magnetic stimulation (TMS), spastic gait, motor-evoked potentials (MEPs), clonus, stiffness

## Abstract

Control of muscles about the ankle joint is an important component of locomotion and balance that is negatively impacted by spinal cord injury (SCI). Volitional control of the ankle dorsiflexors (DF) is impaired by damage to pathways descending from supraspinal centers. Concurrently, spasticity arising from disrupted organization of spinal reflex circuits, further erodes control. The association between neurophysiological changes (corticospinal and spinal) with volitional ankle control (VAC) and spasticity remains unclear. The goal of this scoping review was to synthesize what is known about how changes in corticospinal transmission and spinal reflex excitability contribute to disrupted ankle control after SCI. We followed published guidelines for conducting a scoping review, appraising studies that contained a measure of corticospinal transmission and/or spinal reflex excitability paired with a measure of VAC and/or spasticity. We examined studies for evidence of a relationship between neurophysiological measures (either corticospinal tract transmission or spinal reflex excitability) with VAC and/or spasticity. Of 1,538 records identified, 17 studies were included in the review. Ten of 17 studies investigated spinal reflex excitability, while 7/17 assessed corticospinal tract transmission. Four of the 10 spinal reflex studies examined VAC, while 9/10 examined ankle spasticity. The corticospinal tract transmission studies examined only VAC. While current evidence suggests there is a relationship between neurophysiological measures and ankle function after SCI, more studies are needed. Understanding the relationship between neurophysiology and ankle function is important for advancing therapeutic outcomes after SCI. Future studies to capture an array of corticospinal, spinal, and functional measures are warranted.

## Introduction

Walking is a high-priority goal for most persons with spinal cord injury (SCI) (1). For walking to be the primary means of mobility, several conditions must be satisfied including low energy expenditure, good safety, and adequate speed for practical community-based walking (2). Each of these conditions is highly dependent on a number of factors, including the degree of control present at the ankle (3). Likewise, ankle control is influenced by a multitude of interrelated factors such as muscle strength, timing of activation, and spasticity, which collectively determine gait mechanics (4–6). Without adequate ankle dorsiflexion, foot drop during the swing phase of gait can impair foot clearance, contribute to decreased stride length, and increase likelihood of falls (7). Secondary gait deviations often develop to achieve foot clearance, including excessive hip and knee flexion, limb circumduction, or lateral trunk sway with pelvic hike. These deviations increase the energy cost of walking and decrease the likelihood that walking is a safe and feasible means of mobility (3).

**Figure 1:**
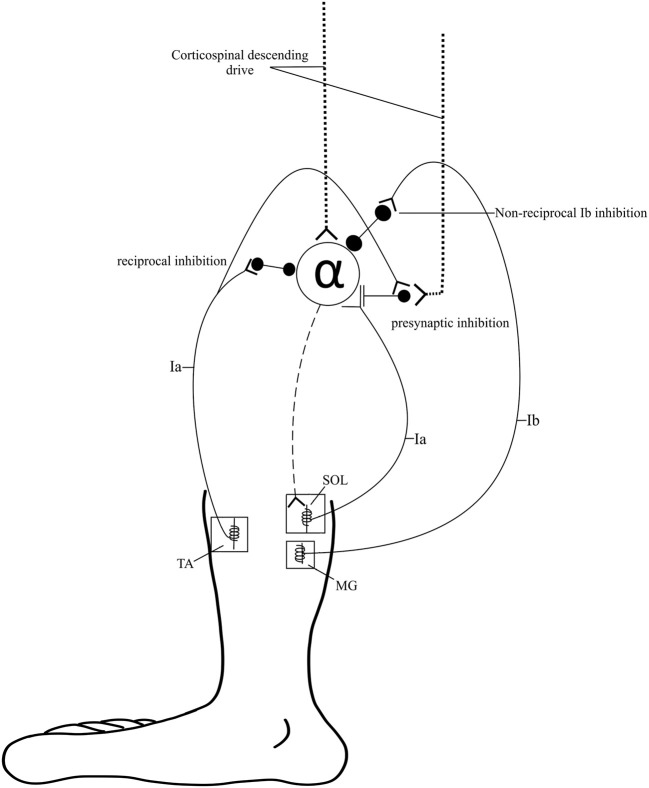
Spinal pathways. Spinal pathways that likely contribute to ankle control and the development of spasticity including reciprocal inhibition, presynaptic inhibition, and non-reciprocal Ib inhibition.

Ankle-related impairments after SCI are attributed to neurophysiologic changes in both corticospinal tract (CST) transmission (8) and modulation of spinal reflex circuit (SRC) excitability (9) (Figure 1). To better understand the influences of CST transmission and SRC excitability, two commonly performed electrophysiological measures are utilized: cortical motor evoked potentials (MEPs) and Hoffman reflex (H-reflex). Both measures are commonly used as non-invasive probes of the underlying neurophysiology of CST transmission and SRC excitability, respectively. Decreased descending transmission impairs volitional control of the dorsiflexors (DF) and reduces the activation of inhibitory inputs to the plantar flexors (PF), further contributing to aberrant SRC activity. It is important to note that while the H-reflex is commonly used as a measure of excitability of the monosynaptic Ia SRCs, this reflex measure reflects oligosynaptic inputs (10).

Some study findings suggest that the reorganization of the cortical motor representation after SCI results in decreased volitional drive through the spared spinal pathways. Evidence indicates that training and neuromodulation approaches directed at increasing volitional drive can increase the amplitude of MEPs (11, 12), restore normal cortical organization (13, 14), and improve volitional muscle activation (15, 16). Altered activity of SRC, due to reorganization of spinal circuits and disruption of normal SRC modulation from descending corticospinal input, can result in several signs and symptoms commonly associated with spasticity after SCI. These symptoms include clonus or hyper-reflexive response to afferent input (i.e., stretch, touch, cold temperatures), muscle stiffness (hypertonia), and spontaneous involuntary muscle contractions (spasms) (17, 18). The maladaptive changes to the circuits controlling the DF and PF following SCI have been described (19–21); however, the relationship between decreased CST descending drive and disrupted SRC modulation with volitional ankle control (VAC) (dorsiflexion during gait, toe tapping, etc.) and/or spasticity remains unclear.

In order to improve functional outcomes after SCI, several recent advances have focused on neuromodulation of the corticospinal and spinal circuits. These advances have been summarized in a recent review (22), and include non-invasive stimulation of afferent inputs such as peripheral nerve somatosensory stimulation, whole body vibration, and transcutaneous spinal cord stimulation. These modalities directly modulate SRC excitability and indirectly activate corticomotor circuits (23). There are also techniques that directly target increased CST descending drive such as transcranial direct current stimulation and repetitive TMS (24). All of these neuromodulatory approaches have been shown to improve functional outcomes, including walking function, when used as an adjuvant to therapy (23). Although these advances show promise for improving walking, to truly optimize functional outcomes it is necessary to understand how neuromodulation of the CST and the SRCs impact variables that are elemental to walking function, such as ankle control and spasticity. As a precursor to exploring the impact of neuromodulation, a better understanding of how neurophysiological measures are related to VAC and spasticity is needed.

To elucidate the respective roles of CST transmission and SRC excitability in disrupted ankle control, we conducted a scoping review to summarize what is known and to identify existing gaps in the literature in order to frame more precise questions for future studies (25). The objectives were to (1) summarize the state of the literature (study designs, methods, evidence of an association), (2) identify existing gaps (variability, contradictions, lack of evidence), and (3) propose future directions (based on existing gaps). Addressing these objectives is important to understand the relationship between corticospinal and spinal neurophysiological measures in the DF and PF and their association with ankle-related function. A better understanding of this association will (1) facilitate the development of more targeted therapeutic strategies for improving ankle control, (2) refine spasticity management, and (3) enhance quality of life for persons with SCI.

## Materials and Methods

In the current review, we used the five stages of a scoping review outlined by Arksey and O'Malley (26): (1) identify the research question, (2) identify relevant studies, (3) select studies for inclusion, (4) [extract and] chart the data, and (5) collate, summarize, and report the results.

### Inclusion/Exclusion Criteria

To determine the scope and extent of the literature, we used inclusion and exclusion criteria that focused on the association between neurophysiological measures and VAC and/or spasticity measures in persons with SCI. For inclusion, all studies had to include adults (mean age ≥18 years old) with SCI. Studies that compared measures obtained from persons with SCI to individuals with other neurological disorders or non-injured individuals were eligible for inclusion. Studies had to include the H-reflex and/or MEPs as measures of spinal and corticospinal neurophysiological changes, respectively. Peripheral nerve stimulation to evoke H-reflexes and transcranial magnetic stimulation (TMS) to evoke MEPs have been shown to be repeatable and consistent in both the DF and PF muscles (27, 28). Studies had to include at least one of these approaches to be eligible for inclusion in the review. To address the relationship between neurophysiological excitability and ankle functional measures, studies had to include at least one measure of VAC (e.g., ankle strength, ankle tapping, foot drop/toe drag during walking) and/or ankle spasticity (e.g., ankle clonus, ankle stiffness). Studies were excluded if subjects who lacked volitional ankle movement were enrolled or if ankle-specific results were not reported. Only studies published in English were included. Case studies, non-peer reviewed sources (e.g., dissertations, conference abstracts, unpublished data), theoretical simulations or models, and reviews were also excluded from the final review.

### Sources and Search

In consultation with a medical librarian, the following databases were searched for articles published between the time of database inception to April 2018: PubMed (includes MEDLINE), Ovid-Medline, and EBSCO-CINAHL. The search terms were chosen to capture articles that included persons with SCI, spinal or corticospinal neurophysiological testing, and functional testing of the ankle DF or PF. The details of the terms and search combinations are described in Table 1. To restrict the population of interest to SCI, the following search terms were always used in combination with the other terms across all databases: (Spinal Cord Injury [Title/Abstract] OR SCI[Title/Abstract] OR spinal damage [Title/Abstract] OR spine damage [Title/Abstract] OR spine injury [Title/Abstract] OR spinal injury [Title/Abstract]). Syntax was adjusted accordingly for each database.

**Table 1:** Detailed search terms.

**Search category**	**Search terms**
SCI	(Spinal Cord Injury [Title/Abstract] OR SCI [Title/Abstract] OR spinal damage [Title/Abstract] OR spine damage [Title/Abstract] OR spine injury [Title/Abstract] OR spinal injury [Title/Abstract])
CST	(Corticospinal Excitability OR CST OR Corticospinal OR corticospinal descending drive OR corticospinal OR TMS OR transcranial magnetic stimulation)
SPINAL	(Spinal reflex circuit OR reflex OR Hoffmann reflex OR H-reflex OR hyperreflexia OR hypertonia OR spinal reflex OR stretch reflex OR monosynaptic reflex)
DF	Control of ankle dorsiflexor OR ankle OR dorsiflexor OR tibialis anterior OR TA OR walking/physiology [MeSH Terms] OR Ankle joint/physiopathology [MeSH Terms] OR Ankle joint/innervation [MeSH Terms] OR Exercise therapy/methods [MeSH Terms] OR Gait Disorders [MeSH Terms] OR Gait [MeSH Terms])
PF	([Control of ankle plantar flexors OR ankle OR plantar flexors OR soleus OR walking/physiology [MeSH Terms] OR Ankle joint/physiopathology [MeSH Terms] OR Ankle joint/innervation [MeSH Terms] OR Exercise therapy/methods [MeSH Terms] OR Gait Disorders [MeSH Terms] OR Gait [MeSH Terms]])

*The initial search term categories were combined in the following ways across 3 databases: 1. SCI + CST+ Spinal+ DF+PF, 2. SCI + CST + DF, 3. SCI + CST + PF, 4. SCI + Spinal + DF, and 5. SCI + Spinal + PF*.

### Screening/Extraction

Article screening was performed using an iterative approach, with 3 screeners (JMH, RZK, and SPE) participating in article selection and review. During the initial screen, the reviewers did not discuss the identity of the articles being considered for inclusion until the end of each screen. At least two of the screeners had to select each study for it to be included in the subsequent screen. There were three total screens: (1) title and abstract, (2) full-text, and (3) full-text with data extraction. For the title and abstract screen, authors only had access to the titles and abstracts to determine relevant studies. During the title and abstract screen, all authors were instructed to examine the text for population, neurophysiological tests of SRC excitability and/or CST transmission, and measures of VAC and/or spasticity. The neurophysiological tests of SRC excitability included measures of H-reflex modulation: reciprocal inhibition, presynaptic inhibition, low-frequency depression, Ib inhibition, ratio of the maximum H-reflex to maximum direct motor response (H/M ratio), and cutaneomuscular -conditioned soleus H-reflexes. Neurophysiologic tests of CST transmission included MEP amplitude and latency. The VAC studies included functional measures such as: DF and PF strength, foot clearance during walking, tapping task, active range of motion (the range of joint movement through which the subject is able to volitionally move the ankle), walking distance, and walking speed. Spasticity studies included biomechanical measures of stretch-induced spastic responses such as: clonus duration, number of oscillations during clonus, and PF reflex threshold angle. If the abstract met all the inclusion criteria, then it was included in the full-text screen. For the full-text screen, authors assessed whether each study met inclusion criteria by skimming through each article once. Finally, during data extraction, the authors determined the relevance of the articles in a more in-depth manner by carefully reading the selected text, while simultaneously extracting specific information from each article. The following information was extracted from each article: study design, study aims, population, participant injury severity, neurophysiological tests, VAC and/or spasticity assessments, and the relationship, if any, between the last two measures. A hand search was conducted on citations in relevant review articles to identify additional articles during the first two screens, and on the articles assessed during the data extraction screen. During the final screen, review articles were excluded.

All articles selected for the final inclusion were grouped based on whether SRC excitability or CST transmission was assessed. The articles were further grouped by whether contributing authors utilized measures of VAC or measures of spasticity. Some articles **directly** determined the relationship between neurophysiological measures and VAC and/or spasticity via correlation or linear regression analyses. In other articles there was no formal testing of the relationship between neurophysiological measures and VAC and/or spasticity. These latter articles were defined as having an **indirect** relationship.

## Results

### Overview of Included Articles

**Figure 2:**
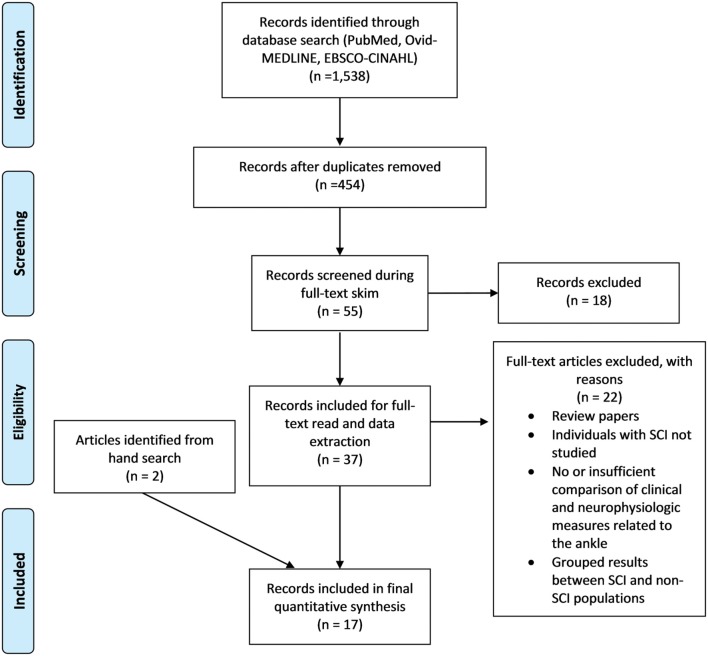
PRISMA flow diagram. PRISMA flow diagram of the Screening process followed during the scoping review (29). For more information, visit www.prisma-statement.org.

In total, 1,538 records were identified in the database searches. After duplicates and dissertations were removed, 454 articles remained, which were subjected to a title and abstract screen. Fifty-five articles were read in full following the title and abstract screen and 18 additional articles were removed for not meeting all inclusion criteria. The remaining 37 articles were assessed for eligibility during the data extraction phase, 22 of which were eliminated for being reviews or not meeting criteria. In addition to the 15 remaining articles, two articles were identified for inclusion during the hand search, bringing the total included article count to 17. Of the 17 included articles, seven had interventional study designs, 10/17 contained measures to assess the relationship between SRC excitability and some aspect of VAC and/or spasticity, and 7/17 articles contained measures of the relationship between CST transmission and VAC. The screening process is illustrated in Figure 2.

The final 17 articles included in this review were published between 1994 and 2017. Neurophysiological data from 277 participants with SCI were captured across all studies. Some participants may be represented more than once, as some lead authors had multiple manuscripts included: Barthélemy (2 articles) (8, 30), Manella (3 articles) (6, 15, 31), and Wirth (4 articles) (32–35). The number of SCI subjects per study ranged from 7 to 40 (median = 15).

#### Demographics of Subjects Enrolled in Included Studies

The majority of subjects had chronic SCI with an initial onset ≥12 months prior to study enrollment. Two of the 17 studies included only subjects with acute or subacute SCI (34, 36). Three studies included subjects with subacute or chronic SCI (32, 33, 35). Of studies that reported subject sex, all had an equal or larger proportion of men to women. While all studies included subjects with motor-incomplete SCI, six also included subjects with motor-complete SCI (6, 37–41). Most studies included a control group of non-injured or “healthy” participants (12/17) (8, 30–32, 34–38, 40, 42, 43), while two studies also included subjects with hemiplegia (32, 37). Participant demographics for each included article are illustrated in Table 2.

**Table 2:** Study characteristics and participant demographics.

**SRC or CST**	**References**	**Study aims**	**ISNCSCI grades**	**Clinical description**	**Mean TPI**	**SCI subject sample size (sex distribution)**	**Mean Age of SCI subjects (y)**	**Non-SCI subject sample size (sex distribution)**	**Mean Age of Non-SCI subjects (y)**
**SRC**									
	Manella et al. (6)	Examine reliability and construct validity of drop test to quantify ankle clonus in persons with SCI and compare results with the SCATS and H/M ratio	A–D	C2 to L1 LOI, Evidence of clonus	8.9 y	*n* = 40 (31 M, 9 F)	39.6 ± 13	N/A	N/A
	Piazza et al. (36)	Examine the effects of leg-cycling on conditioned H-reflex excitability and how it relates to lower extremity function after miSCI	C to D	C5-T10 LOI, capable of cycling	16 ± 3 weeks	*n* = 9 (6 M, 3 F)	44 ± 5	Non-injured: *n* = 10	34 ± 3
	Yamaguchi et al. (43)	Compare effect of anodal tDCS and PES on RI and ankle movement in miSCI	C to D	C1 to T11, 1^+^ PF spasticity (MAS)	4.5 ± 4.2 y	*n* = 11 (11 M)	51.8 ± 10.7	Non-injured: *n* = 10	50.7 ± 8.9
	Smith et al. (41)	Assess changes in H-reflex in different positions after locomotor training in persons with SCI	A to D	C1 to T10 LOI	4.3 y	*n* = 15 (10 M, 5 F)	37.53	N/A	N/A
	Manella and Field-Fote (31)	1. Investigate effects of LT on measures of spasticity and walking. 2. Assess association of change in walking speed with measures of reflex activity 3. Establish sensitivity to change and validity of PF RTA	C or D	C4 to L1 LOI, Evidence of clonus in some participants	8.9 y	Locomotor cohort: *n* = 18 (16 M, 2 F) Validity cohort: *n* = 40 (30 M, 10 F)	Locomotor cohort: 35.1 Validity cohort: 39.3	Non-injured: *n* = 10	27.3
	Manella et al. (15)	1. Examine effects of different operant conditioning interventions on ankle motor control, spasticity, and walking related measures in persons with miSCI. 2. Explore relationship between changes in neurophysiological and clinical outcome measures.	D	Positive ankle clonus, Median LL of TA group: C7; Median LL of SOL group: C5	TA group: 10.8 ± 10.0 y; SOL group: 10.8 ± 08.8 y	Total: *n* = 12; TA cohort: *n* = 6 (6 M); SOL cohort: *n* = 6 (4 M, 2 F)	TA cohort: 44.2; SOL cohort: 45.2	N/A	N/A
	Adams and Hicks (39)	Examine effects of BWSTT and TTS on spasticity and motor neuron excitability in chronic SCI	A to C	C5 to T10 LOI, stable spasticity, primary wheelchair user	5.0 ± 4.4 y	*n* = 7 (6 M, 1 F)	37.1 ± 7.7	N/A	N/A
	Murillo et al. (40)	Examine the effect of RF vibration on clinical and neurophysiological outcome measures of spasticity in SCI	A to D	C3 to T11 LOI; lower limb spasticity ≥ 1.5 (MAS)	5.6 y ± 1.9 months	*n* = 19 (16 M, 3 F)	36.0 ± 10.6	Non-injured: *n* = 9	33.8 ± 9.4
	Downes et al. (38)	Examine reflex actions of MG group Ib afferent stimulation on SOL H-reflex excitability and spasticity in persons with SCI	Both cSCI and iSCI	C4 to T10 LOI	16 mos	*n* = 13 (11 M, 2 F)	30	Non-injured: *n* = 20	25
	Faist et al. (37)	Assess effect of femoral nerve stimulation on SOL H-reflex activity in SCI. Examine association of spasticity and PI	Both cSCI and iSCI	LOI not reported	27.5 mos	*n* = 17	34.8	Non-injured: *n* = 28, Hemiplegic: *n* = 18	Non-injured age range: 21-59; Hemiplegic mean age: 49.6
**CST**									
	Barthélemy et al. (30)	Examine the correlation of CST function and measures of gait and ankle function after SCI	D	C1 to L1 LOI	12 y	*n* = 24 (22 M, 2 F)	43.4	Non-injured: *n* = 11	45
	Labruyère et al. (42)	Assess deficits in quick and accurate movements in miSCI by combining TMS, EMG, and a response time task and comparing differences in clinical characteristics.	D	T2 to L4 LOI only	6.3 ± 5.5 y	n = 15(10 M, 5 F)	50.2 ± 12.4	Non-injured: *n* = 15	50.1 ± 12.3
	Barthélemy et al. (8)	Examine the relationship between parameters that may reflect CST function and physical foot drop deficit observed after SCI	D^+^	C to L LOI; ability to walk 10m	12 ± 11 y	*n* = 24 (22 M, 2 F)	43 ± 14	Non-injured: *n* = 15	42 ± 16
	Wirth et al. (35)	Examine ankle DF timing during gait and in supine to CST conductivity and measures of strength and gait speed in persons with and without SCI.	C or D	C2 to T12	2.7 ± 3.5 y	*n* = 12 (9 M, 3 F)	58.3 ± 10.7	Non-injured: *n* = 12	59.2 ± 11.3
	Wirth et al. (32)	Examine the effects of CST damage on ankle dexterity and MMV in individuals with miSCI and stroke	miSCI	C3 to L5 LOI	13.3 ± 31.7 mos	*n* = 12 (6 M, 6 F)	62.3 ± 8.3	Stroke: *n* = 12; Non-injured: *n* = 12	Stroke 65.8 ± 10.5; Non-injured: 63.3 ± 10.7
	Wirth et al. (33)	Examine the relationship between ankle dorsiflexor strength, MVC, and MMV with CST integrity and with walking capacity in persons with miSCI	C or D	C3 to L1 LOI	Strength ^+^ MEP cohort: 2.4 ± 3.5 y; strength ^+^ gait cohort: 1.0 ± 2.2 y	*n* = 26; strength ^+^MEP *n* = 17 (14 M, 3 F); strength ^+^ gait *n* = 19 (14 M, 5 F)	Strength ^+^ MEP cohort: 50.8 ± 16.5; strength ^+^ gait speed cohort: 54.3 ± 15.2	N/A	N/A
	Wirth et al. (34)	Examine recovery of ankle DF in miSCI via neurophysiological assessment of CST function and functional parameters	C or D	C3 to T12 LOI	All subjects tested at 1,3, and 6 mos post-injury	*n* = 12 (6 M, 6 F)	53.7 ± 18.5 months	Non-injured: n = 12	54.0 ± 18.0

+ *One subject lacked sacral sparing and had an AIS A classification, but had motor function equivalent to AIS D*.

*SRC, Spinal Reflex Circuitry; CST, corticospinal tract*.

*^a^ISNCSCI, International Standards for Neurological Classification of Spinal Cord Injury; TPI, time post-injury; SCI, spinal cord injury; SCATS, Spinal Cord Assessment Tool for Spastic Reflexes; LOI, level of injury; N/A, not applicable; miSCI, motor incomplete spinal cord injury; tDCS, transcranial direct current electrical stimulation; PES, patterned electrical stimulation; RI, reciprocal inhibition; MAS, modified Ashworth scale; LT, locomotor training; PF, plantar flexor; RTA, reflex threshold angle; LL, lesion-level; TA, tibialis anterior; SOL, soleus; BWSTT, body-weight supported treadmill training; TTS, tilt table standing; RF, rectus femoris; cSCI, complete spinal cord injury; iSCI, incomplete spinal cord injury; MG, medial gastrocnemius; PI, presynaptic inhibition; TMS, transcranial magnetic stimulation; EMG, electromyography; MMV, maximal movement velocity; MVC, maximal voluntary contraction; MEP, motor evoked potential; DF, dorsiflexion; y, years*.

#### SRC Excitability Articles

There were 10 studies that examined changes in SRCs. Four of 10 also included measures of VAC (15, 36, 38, 43); all four included correlation and/or association analyses to quantify the relationship between SRCs and VAC. Nine of 10 studies with SRC measures also had measures of ankle spasticity (6, 15, 31, 36–41); only six of these articles included correlation and/or association analyses to quantify the relationship between SRC excitability and spasticity (6, 15, 31, 36–38). There were 3/10 SRC articles that assessed both VAC and spasticity (15, 36, 38).

#### CST Transmission Articles

There were seven studies that examined changes in CST function, all of which included measures of VAC (8, 30, 32–35, 42). Six of seven performed correlation and/or association analyses to quantify the relationship between CST function and VAC (8, 30, 32–35). One study of CST transmission included subjects with stroke in addition to subjects with SCI without separating the data of those individuals prior to performing linear regression analysis, making it difficult to parse out SCI results from results of participants with stroke (32). None of the studies examining CST transmission included measures of ankle spasticity.

### Relationship Between SRC Excitability and VAC

Disrupted modulation of SRC excitability after SCI has been associated with decreased VAC. This impaired ability to voluntarily activate ankle DF can negatively impact walking function (8). In the current review, 3/4 studies that included measures of SRC excitability and VAC (Table 3) provide evidence of a direct association between these measures (15, 36, 43). All three studies that support a direct relationship between SRC excitability and VAC had interventional study designs (15, 36, 43). Among the VAC measures included in these studies were foot clearance, foot tapping, DF strength, PF strength, active range of motion, and walking distance over time. Although evidence of an association between SRC excitability and VAC is clear, there was a wide range of SRC measures and interventions used across these studies (Table 3).

**Table 3:** Is there a relationship between SRC excitability and volitional ankle control?

**References**	**Study Design**	**Intervention**	**Electrophysiological measure**	**Relevant functional measure**	**Direct or indirect evaluation of association?**	**Evidence of association?**
Piazza et al. (36)	Interventional	Leg-cycling	Plantar cutaneomuscular conditioned SOL^a^ H-reflex	Strength (TA and Triceps Surae manual muscle score)	**Direct** (Multiple Stepwise Forward Regression and Spearman's Correlation)	Yes
Yamaguchi et al. (43)	Interventional	Anodal tDCS combined with PES	SOL H-reflexes in response to RI and PI	Ankle movement/tapping, Active ankle ROM	**Direct** (Pearson's correlation)	Yes
Manella et al. (15)	Interventional	Operant conditioning: TA EMG activation increase OR SOL H-reflex decrease	SOL H-reflexes in response to RI, PI, and LFD	Toe/foot clearance during walking, ankle movement/tapping, strength (DF and PF), active ROM (DF)	**Direct** (Spearman's correlation)	Yes
Downes et al. (38)	Observational	N/A	Ib conditioned-SOL H-reflex	Strength (DF and PF)	**Direct** Pearson's correlation	No

a *SOL, soleus; TA, tibialis anterior; tDCS, transcranial direct current stimulation; PES, patterned electrical stimulation ; RI, reciprocal inhibition; PI, presynaptic inhibition; ROM, active range of motion; EMG, electromyography; LFD, low frequency depression; DF, dorsiflexor; PF, plantar flexor*.

*“Yes” indicates that there is evidence that at least one electrophysiological measure had an association with at least one relevant functional measure*.

In contrast to the three interventional studies that provided evidence of a relationship between SRC excitability and VAC, the observational study captured during this review (38) assessed Ib inhibition from the medial gastrocnemius onto the soleus H-reflex. The authors concluded that Ib inhibition is not affected by SCI. This may suggest that the descending spinal circuits that modulate Ib SRCs differ from those that modulate Ia SRC excitability.

### Relationship Between SRC Excitability and Ankle Spasticity

The development of spasticity in the ankle PFs is associated with poorer functional outcomes, interfering with ability to walk and perform daily tasks such as transfers (17). Of the nine studies with measures of ankle spasticity (Table 4), six provided evidence of an association with SRC excitability (6, 15, 31, 36, 40, 41). Only four of those six studies used statistical tests to quantify the relationship between SRC excitability and spasticity (6, 15, 31, 36). Five of the six studies that provided evidence of an association had an interventional design (15, 31, 36, 40, 41). Overall, these six studies had a large spectrum of interventions, assessments of spasticity, and measures of SRC excitability (Table 4).

**Table 4:** Is there a relationship between SRC excitability and ankle spasticity?

**References**	**Study design**	**Intervention**	**Electrophysiological measure**	**Relevant functional measure**	**Direct or indirect evaluation of association?**	**Evidence of association?**
Faist et al. (37)	Observational	N/A	PI (Quadriceps), SOL H/M ratio	Hypertonia (Achilles tendon reflex, Ashworth scale)	**Direct** (Pearson's correlation)	No
Downes et al. (38)	Observational	N/A	Ib conditioned-SOL H-reflex	Hypertonia (Achilles tendon reflex; tone of the ankle DF, PF)	**Direct** (Pearson's correlation)	No
Adams and Hicks (39)	Interventional	BWSTT v. TTS	SOL H/M ratio	Clonus duration (SCATS-Clonus)	**Indirect**	No
Murillo et al. (40)	Interventional	Focal vibration (RF)	SOL H/M ratio	Clonus duration and # of oscillations	**Indirect**	Yes
Manella and Field-Fote (31)	Interventional AND Observational	Locomotor training	SOL H/M ratio	Clonus duration, # of oscillations, PF RTA (Drop test), gait speed	**Direct** (Spearman's correlation)	Yes
Manella et al. (15)	Interventional	Operant conditioning: TA activation increase OR Soleus H-reflex suppression	SOL H-reflex: RI, PI, and LFD	Clonus duration, PF RTA	**Direct** (Spearman's correlation)	Yes
Manella et al. (6)	Observational	N/A	SOL H/M ratio	# of oscillations (Drop test)	**Direct** (Spearman's correlation)	Yes
Smith et al. (41)	Interventional	Locomotor training (Lokomat)	SOL H/M ratio	Clonus duration and # of oscillations (via soleus EMG analysis during walking)	**Indirect**	Yes
Piazza et al. (36)	Interventional	Leg-cycling	Plantar^a^ cutaneomuscular conditioned SOL H-reflex	Hypertonia (MAS), clonus duration (SCATS clonus score)	**Direct** (Multiple Stepwise Forward Regression and Spearman's Correlation)	Yes

a *PI, presynaptic inhibition; SOL, soleus; DF, dorsiflexor; PF, plantar flexor; BWSTT, body-weight supported treadmill training; TTS, tilt table standing; SCATS, Spinal Cord Assessment Tool for Spastic Reflexes; RF, rectus femoris; RTA, reflex threshold angle; EMG, electromyography; RI reciprocal inhibition; LFD, low frequency depression*.

*“Yes” indicates that there is evidence that at least one electrophysiological measure had an association with at least one relevant functional measure*.

Additionally, the two studies that did not support an association between measures of spasticity and measures of SRC excitability were observational (Table 4) (37, 38). One of these studies was described above in the section on VAC (38), wherein Ib inhibition from the medial gastrocnemius onto the soleus H-reflex was not found to be influenced by SCI. The authors likewise concluded that there was no relationship between excitability of this circuit and the Achilles tendon reflex testing. The other study (37) assessed the level of heteronomous Ia facilitation between the quadriceps and the soleus H-reflex amplitude as an index of presynaptic inhibition. The authors concluded that while there was less presynaptic inhibition in those with SCI, there was no relationship between the amount of presynaptic inhibition and the Ashworth scale scores. This conclusion directly conflicts with studies that have assessed presynaptic inhibition in other circuits and concluded that decreased presynaptic inhibition is associated with spasticity (44, 45).

Of the 6 interventional studies with assessments of SRC excitability and ankle spasticity, only one did not provide evidence of an association (39). In this study, clonus duration decreased more after body weight supported treadmill training compared to standing on a tilt table; however, there was no change in H/M ratio associated with either intervention. The lack of change in H/M ratio may be due to methodological issues, as prior studies have shown that the maximum H-reflex is less sensitive to modulatory influence than are submaximal reflex responses.

### Relationship Between CST Transmission and VAC

Damage to the CSTs associated with SCI has been associated with deficits in walking ability and balance (46). There is evidence that CST transmission is also related to VAC in all seven of the articles in which the relationship between CST transmission and VAC was assessed (Table 5).

**Table 5:** Is there a relationship between CST transmission and volitional ankle control?

**References**	**Study design**	**Intervention**	**Electrophysiological measure**	**Relevant functional measure**	**Direct or indirect evaluation of association?**	**Evidence of association?**
Barthélemy et al. (30)	Observational	N/A	TA MEP^a^ amplitude, latency	Gait kinematics toe clearance, gait speed, walking distance	Direct (Spearman's and Pearson's correlation)	Yes
Labruyère et al. (42)	Observational	N/A	MEP amplitude, latency	Muscle strength (DF and PF), stepping task	Indirect	Yes
Barthélemy et al. (8)	Observational	N/A	TA MEP amplitude, latency	Gait kinematics – foot drop	Direct (Spearman's and Pearson's correlation)	Yes
Wirth et al. (35)	Observational	N/A	MEP amplitude, latency	Timing of ankle dorsiflexion during gait and in supine at 3 frequencies, DF MMV, TA muscle strength (MVC)	Direct and Indirect (Spearman's correlation)	Yes
Wirth et al. (32)	Observational	N/A	TA MEP amplitude, latency	Ankle dexterity, MMV	Simultaneous Direct (linear regression analyses-backward standardized regression GROUPED TOGETHER)	Yes
Wirth et al. (33)	Observational	N/A	MEP amplitude, latency	TA muscle strength (AIS motor score, MVC), DF MMV, gait speed, walking ability (WISCI)	Direct (linear, backwards multiple regression)	Yes
Wirth et al. (34)	Observational	N/A	MEP amplitude, latency	ankle dexterity, MMV, TA strength (AIS motor score, MVC), gait speed	Direct (linear regression and Spearman's correlation)	Yes

a *TA, tibialis anterior; MEP, motor evoked potential; DF, dorsiflexor; PF, plantar flexor; MMV, maximal movement velocity; MVC, maximal volitional contraction; AIS, ASIA impairment scale; WISCI, Walking Index for Spinal Cord Injury*.

*“Yes” indicates that there is evidence that at least one electrophysiological measure had an association with at least one relevant functional measure*.

In contrast to the SRC studies, none of the CST transmission studies used an intervention; all had observational designs. Six of seven studies used direct measures of correlations or linear regressions to assess the relationship between CST transmission and volitional measures (8, 30, 32–35). Seven of 7 studies assessed MEP amplitude and latency in the TA. Functional measures including foot clearance, maximal movement velocity of the ankle, walking speed, walking distance over time, timing of dorsiflexion during walking, and ankle strength were all related to CST transmission. In one of these studies, a prospective cohort design was used to record longitudinal changes in the first six months after SCI. MEP amplitude, gait speed, DF muscle strength, and rate of activation increased significantly over time (34).

## Discussion

### Summary—State of the Literature

While empirical evidence suggests there is an association between (1) measures of SRC excitability and VAC, (2) SRC excitability and spasticity, and (3) between CST transmission and VAC, the relationship between these measures in the literature is confounded by inherent variability in the neurophysiological measures and the wide range of functional measures utilized across the studies. To gain a better understanding of the evidence that does exist regarding the relationship between the underlying neurophysiological tests and ankle control, biomechanical, and functional outcomes were more closely examined and compared to specific neurological tests within and between studies (Tables 3–5).

#### Relationship Between SRC Excitability and VAC

After SCI, control of dorsiflexion depends on the extent to which hyperexcitability of the soleus SRCs degrades normal ankle kinematics. Ankle control can be examined using a variety of functional measures. It is important to determine the underlying mechanisms involved with each of these measures, as this knowledge could support the development of more effective rehabilitation strategies. The findings of this scoping review provide evidence that different components of ankle control may be associated with distinct measures of SRC excitability.

For the measure of ankle control during tapping tasks, 2 studies (15, 43) support the conclusion that the number of repetitions of ankle movements during a timed tapping task is associated with H-reflex excitability as measured by presynaptic and reciprocal inhibition. The relationship between ankle strength and reflex excitability is less clear, as two studies indicate there could be an association between strength and amplitude of the conditioned H-reflex responses (15, 36), while another did not (38). In addition to the divergence of findings, and perhaps the reason for the divergence, the type of H-reflex test used for each of these studies varied (see Table 3). For the two studies that measured active range of motion in the ankles, the first study demonstrated a possible association between this functional outcome with low frequency depression and presynaptic inhibition (15), while the other demonstrated a change in reciprocal and presynaptic inhibition, but not active range of motion (43). Lastly, in the study in which toe clearance during walking was measured, there was an association between change in toe clearance and reciprocal inhibition (15). Although most of these studies included correlations between SRC excitability and VAC measures, due to the variability of the tests, there is a strong need for additional studies to quantify the relationship between these two constructs.

#### Relationship Between SRC Excitability and Ankle Spasticity

The ability to achieve adequate dorsiflexion during functional tasks, such as walking and transfers, can be hindered by involuntary muscle contractions and stiffness associated with spasticity in the ankle plantar flexors. It is important to understand how commonly used tests of SRC excitability are associated with biomechanical measures of spasticity to develop more effective neuromodulatory strategies. In comparison to the number of studies that included measures of SRC excitability and VAC, there is a noticeably larger amount of studies dedicated to measuring SRC excitability and ankle spasticity. There are several biomechanical assessments to measure ankle spasticity, including: spinal cord assessment tool for spastic reflexes (SCATS), modified Ashworth scale, Achilles tendon reflex, and the ankle clonus drop test. These biomechanical tests can provide insight into properties such as ankle stiffness, clonus duration, and number of clonus oscillations. The large variety of biomechanical spasticity measures used across studies and the different types of SRC excitability measures utilized, make it difficult to quantify the relationship between the biomechanical measures of responsiveness to stretch and electrophysiologic SRC excitability measures. However, there is evidence that some biomechanical measures of spasticity may be associated with different components of SRC excitability (see Table 4).

One sensitive biomechanical measure of spasticity is the reflex threshold angle in the plantar flexors. One study provided evidence that reflex threshold angle appears to be related to reciprocal and presynaptic inhibition (15), while another showed it may be related to H-reflex excitability (31). Of the 6 studies that included measures of clonus duration, three provide evidence that there may be a relationship between clonus duration and cutaneomuscular conditioned-reflex (36), H-reflex excitability (40), and low frequency depression (15). The same number of articles demonstrated a potential association between number of oscillations during ankle clonus and H-reflex excitability (6, 40, 41). Only one study demonstrated evidence of a relationship between ankle stiffness, measured using the modified Ashworth scale, and cutaneomuscular conditioned-reflex (36). Though there is some evidence of an association between measures of SRC excitability and functional outcomes related to spasticity, an increased amount of attention into the specifics of these measures and the underlying mechanisms that impact them is needed for a better understanding of this relationship.

#### Relationship Between CST Transmission and VAC

Spinal cord injury diminishes the capacity of the CST to transmit descending neural signals, thereby limiting both strength and speed of VAC in the dorsiflexors. Measures of VAC included: tapping tasks, ankle strength, toe clearance during walking, and gait measures. Both MEP amplitude and latency were shown to have some evidence of a relationship with each component of ankle control (see Table 5). It should be noted that although there were some differences in the methodologies used across studies, there was less variability between measures in the CST transmission studies than there were in the SRC excitability studies.

The coordination and timing of VAC can be assessed during tapping tasks to match a rhythmic tone. In the four studies that assessed VAC during tapping tasks, there was an association between maximal movement velocity and measures of CST transmission (32–35). Ankle strength is another important component of VAC. For the measure of ankle strength, two articles demonstrated an association between strength with MEP latency (33) and MEP amplitude (34), while two other articles did not support a relationship between those measures (35, 42). Foot drop/toe drag can be assessed by measuring toe clearance and ankle angle during swing phase. Toe clearance during walking was measured in two articles that assessed CST transmission (8, 30). Both articles demonstrated that maximum toe elevation was associated with MEP amplitude and latency. Lastly, 4/6 studies that contained some measure of gait parameters and stepping ability, presented evidence of an association with CST neurophysiology (8, 30, 35, 42). Additional work is warranted in this area to understand how measures of CST transmission relate to ankle spasticity, as none of these studies included any spasticity measures. There would be great value in future studies that include CST transmission and SRC excitability measures in conjunction with ankle-related functional and biomechanical outcomes.

### Existing Gaps—Limitations

#### Limitations of Included Studies

The greatest limitation in the currently available literature related to the relationships among CST transmission, SRC excitability, spasticity, and function was the large variability in measures used in the studies. Overall, there was a wider range of neurophysiological measures in the studies that assessed SRC excitability than in the studies that assessed CST transmission. Although all of the reported neurophysiological measures assessed changes in SRC excitability, the studies tested different circuits at different timepoints, which may result in significant changes being observed in one study while non-significant results were observed in another. For example, although presynaptic inhibition and reciprocal inhibition both impact reflex excitability, the interneurons involved are not the same. Given that changes in the CST transmission can influence the SRC excitability, it is unfortunate that no articles included both corticospinal and spinal neurophysiological measures.

There was also variability in the types of measures used to assess spasticity and VAC in the studies which assessed SRC excitability and the articles that assessed CST transmission. In the articles that addressed SRC excitability, the studies included different biomechanical measures of ankle spasticity. None of the included CST transmission articles assessed spasticity. Both measures are important for assessing factors that influence changes in gait parameters after injury. Increases in voluntary control and decreases in spasticity are both beneficial for improving walking function in persons with SCI. Future studies should assess both volitional and spasticity related measures of the ankle.

#### Limitations of Review

The search strategy was potentially limited for two main reasons. The search included only studies of SRC excitability that used measures based on the H-reflex test and studies of CST transmission that used MEPs as neurophysiological measures. Studies which utilize other neurophysiological measures along with VAC and spasticity measures may have been excluded, potentially limiting the scope of this review. We chose to use H-reflex and MEPs as the primary measures of interest because they are both widely used, non-invasive neurophysiological tests with good reliability. However, despite being commonly used measures, H-reflexes and MEPs cannot isolate or provide information about the integrity of all pathways that may influence neuromotor control of the ankle (i.e., rubrospinal tract, reticulospinal tract, vestibulospinal tract, and group II afferent nerve pathways).

### Conclusions and Future Directions

Based on the available literature there is evidence of an association between neurophysiological excitability with VAC and spasticity after SCI. Future studies assessing these relationships are important for the development of better targeted therapies such as whole body vibration, peripheral nerve somatosensory stimulation, transcutaneous spinal stimulation, and transcranial direct current stimulation to improve walking and balance in individuals affected by SCI. There is great potential for this knowledge to guide therapists in the use of non-invasive stimulation to increase descending drive or decrease spasticity. While it may be difficult to isolate interventions to either CST transmission or SRC excitability alone, it is important to understand neurophysiologic contributions to ankle control given its relevance to safe and efficient ambulation within clinical populations with central nervous system disorders. Studies that employ a battery of neurophysiologic and functional measures to assess both SRC excitability and CST transmission in persons with SCI are warranted.

## Author Contributions

JH was responsible for the development of the review question, conduction of the literature search, screening process, and wrote a substantial portion of each section. RK assisted with the screening process, created the tables and figures, and wrote portions of the background, results, and discussion. SE assisted in the screening process and developed sections of the results and discussion. EF-F assisted in the development of the review question, was a consultant during each stage of the review process, and played a role in the development and editing of the entire scoping review.

### Conflict of Interest

The authors declare that the research was conducted in the absence of any commercial or financial relationships that could be construed as a potential conflict of interest.

## Abbreviations

SCI: spinal cord injury
DF: ankle dorsiflexors
VAC: volitional ankle control
SRC: spinal reflex circuitry
CST: corticospinal tract
H-reflex: Hoffman reflex
MEP: motor evoked potentials
PF: plantar flexors
TMS: transcranial magnetic stimulation.
